# Genome-Wide Association Study of Cadmium Accumulation at the Seedling Stage in Rapeseed (*Brassica napus* L.)

**DOI:** 10.3389/fpls.2018.00375

**Published:** 2018-04-19

**Authors:** Lunlin Chen, Heping Wan, Jiali Qian, Jianbin Guo, Chengming Sun, Jing Wen, Bin Yi, Chaozhi Ma, Jinxing Tu, Laiqiang Song, Tingdong Fu, Jinxiong Shen

**Affiliations:** ^1^National Key Laboratory of Crop Genetic Improvement, National Engineering Research Center for Rapeseed, College of Plant Science and Technology, Huazhong Agricultural University, Wuhan, China; ^2^Nanchang Branch of National Center of Oilcrops Improvement, Jiangxi Province Key Laboratory of Oil Crops Biology, Crops Research Institute of Jiangxi Academy of Agricultural Sciences, Nanchang, China

**Keywords:** *Brassica napus*, cadmium, genome-wide association study, genotypic variations, hyperaccumulator, quantitative trait loci

## Abstract

Cadmium is a potentially toxic heavy metal to human health. Rapeseed (*Brassica napus* L.), a vegetable and oilseed crop, might also be a Cd hyperaccumulator, but there is little information on this trait in rapeseed. We evaluated Cd accumulation in different oilseed accessions and employed a genome-wide association study to identify quantitative trait loci (QTLs) related to Cd accumulation. A total of 419 *B. napus* accessions and inbred lines were genotyped with a 60K Illumina Infinium SNP array of Brassica. Wide genotypic variations in Cd concentration and translocation were found. Twenty-five QTLs integrated with 98 single-nucleotide polymorphisms (SNPs) located at 15 chromosomes were associated with Cd accumulation traits. These QTLs explained 3.49–7.57% of the phenotypic variation observed. Thirty-two candidate genes were identified in these genomic regions, and they were 0.33–497.97 kb away from the SNPs. We found orthologs of *Arabidopsis thaliana* located near the significant SNPs on the *B. napus* genome, including NRAMP6 (natural resistance-associated macrophage protein 6), IRT1 (iron-regulated transporter 1), CAD1 (cadmium-sensitive 1), and PCS2 (phytochelatin synthase 2). Of them, four candidate genes were verified by qRT-PCR, the expression levels of which were significantly higher after exposure to Cd than in the controls. Our results might facilitate the study of the genetic basis of Cd accumulation and the cloning of candidate Cd accumulation genes, which could be used to help reduce Cd levels in edible plant parts and/or create more efficient hyperaccumulators.

## Introduction

Cadmium is a potentially toxic heavy metal, which has entered agricultural soils via atmospheric deposition and fertilization with manure, phosphates, and sewage sludge (Tanhuanpää et al., 2007). In humans, Cd is ingested via contaminated food, and it is genotoxic and cytotoxic, inhibits cell growth, and induces apoptosis (Rafiq et al., 2014; Touceda-González et al., 2015). Cd is efficiently accumulated in the kidneys, where it has a biological half-life of 10–30 years, and thus, the Cd burden in the body increases with age (Clemens et al., 2013). Plant roots can readily absorb and translocate Cd to the shoots. It is, therefore, necessary for food safety and human health to reduce Cd accumulation in edible plant parts and minimize human dietary Cd intake. Rapeseed (*Brassica napus* L., 2*n* = 38, AACC) is a vegetable and an important source of edible oil. The Cd distribution in rapeseed organs is in the following decreasing order: leaves > stems > roots > pods > seeds (Angelova et al., 2005). In addition, rapeseed Cd content tends to increase with soil Cd levels (Selvam and Wong, 2008). For this reason, it is preferable to select and cultivate rapeseed accessions that accumulate low Cd concentrations.

A report on soil environmental quality in China indicated that 16% of the arable land is polluted, and 7% of it is contaminated specifically with Cd (Song et al., 2016). Several techniques have been used to reduce the Cd levels in paddy fields, including alkaline amendments, water management, and soil dressing (Ishikawa et al., 2005). Phytoremediation enables the detoxification of heavy metals from water, soil, and air (Raskin and Ensley, 1999). In some cases, phytoremediation might be the only effective way to restore land and water polluted by human activities. Plant species selected for phytoremediation must be able to absorb heavy metals in high concentrations in the shoots. *Brassica juncea* (Indian mustard) has been used in phytoremediation as well (Shanmugaraj et al., 2013). Rapeseed is cultivated more extensively and has higher yields than *B. juncea*. Analyses of different rapeseed varieties to extract Cd from the soil have indicated *B. napus* to be an excellent Cd hyperaccumulator. It can extract and concentrate Cd to a level of 0.8–1.22% from heavily contaminated soil (Cojocaru et al., 2016). Nevertheless, there are few reports on the phenotypic variations in rapeseed Cd accumulation; therefore, the evaluation of the Cd concentration capacities of several rapeseed varieties would help assess their phytoremediation potential.

Recently, progress has been made in identifying the genes that mediate Cd accumulation in rice (*Oryza sativa* L.). Differences in Cd root accumulation, root-to-shoot translocation, and transportation to organs account for variations in Cd accumulation among crops (Ueno et al., 2009; Clemens et al., 2013). Detailed molecular genetic maps were constructed and subsequently used to identify quantitative trait locus (QTL) for Cd accumulation in *Arabidopsis halleri*, rice, maize, soybean, and radish (Benitez et al., 2012; Xu et al., 2012; Abe et al., 2013; Zdunić et al., 2014; Meyer et al., 2016). Furthermore, genome-wide association studies (GWAS) have also been performed in *Arabidopsis thaliana* and barley for Cd accumulation (Chao et al., 2012; Wu D. et al., 2015). However, such studies have not been reported in rapeseed.

Several genes involved in Cd accumulation may control plant Cd accumulation. These genes have been sought out using QTL analyses or functional analyses. The gene coding for the natural resistance-associated macrophage protein (NRAMP) is the major contributor to Mn^2+^ and Cd^2+^ translocation and distribution in rice shoots. An *AtNRAMP4* mutation modified Cd^2+^ and Zn^2+^ accumulation in rice without affecting iron transport. Cd^2+^ movement might be limited using NRAMP transporters (Pottier et al., 2015). The knockout of OsNramp5, a transporter in the plasma membrane on the distal root exodermis and endodermis, significantly decreased Cd accumulation in rice grains (Sasaki et al., 2012). OsHMA3, a Cd transporter in the tonoplast, sequestered some of the Cd into root-cell vacuoles (Ueno et al., 2010). Functional OsHMA3 overexpression significantly reduced Cd accumulation in rice grains and increased shoot Cd tolerance without impeding growth. Loss-of-function mutations in *OsHMA3* resulted in high Cd accumulation in rice grains (Ueno et al., 2010; Sasaki et al., 2014). SpHMA3 was found to maintain the normal growth of the young leaves of *Sedum plumbizincicola* plants growing in Cd-contaminated soils (Liu et al., 2017). OsHMA2, a homolog of OsHMA3, mediates the root-to-shoot translocation of Cd (Yamaji et al., 2013). In contrast, little is known regarding the molecular mechanisms of Cd accumulation in rapeseed.

Genome-wide association study has been employed to dissect complex traits in plants such as rice (Schläppi et al., 2017), *A. thaliana* (Chao et al., 2012), and maize (Xiao et al., 2017). GWAS has led to the identification of QTLs for complex rapeseed traits such as flowering time (Xu et al., 2015), oil content (Liu et al., 2016), branch angle traits (Sun et al., 2016a), salt tolerance-related traits (Wan et al., 2017), erucic acid content, glucosinolate content, seed weight, plant height, primary branch (Li et al., 2014, 2016), pod shatter resistance (Raman et al., 2014), and blackleg resistance (Raman et al., 2016). Previous research showed that GWAS costs less time because, unlike in linkage mapping, a mapping population need not be constructed, and the identification of genetic loci is also more efficient than when using linkage mapping (Liu et al., 2016; Xiao et al., 2017). However, GWAS for the QTLs of Cd accumulation traits has only been reported in *A. thaliana* and barley (Chao et al., 2012; Wu D. et al., 2015). Very few QTLs for Cd accumulation in rapeseed have been studied.

In the present study, we examined 419 global rapeseed accessions using 19,167 genomic single-nucleotide polymorphisms (SNPs) obtained by the Illumina Brassica SNP60 Bead Chip and investigated the genotypic variations in Cd accumulation at the seedling stage in plants raised in three different cultivation environments. We then performed a GWAS for Cd accumulation and detected several QTLs controlling this trait.

## Materials and Methods

### Core Rapeseed Accessions

The test population consisted of 419 genetically diverse inbred lines representing three types of oilseed rape (OSR) germplasm: (a) winter OSR (29), (b) semi-winter OSR (355), and (c) spring OSR (35) (Supplementary Table S1), which were used in a recently published study (Xu et al., 2015). China contributed to 383 accessions originating from three rapeseed subregions with diverse climate, soil fertility, and hydrology. They broadly represent the genetic diversity of the Chinese rapeseed gene pool.

### Hydroponic Experiments and Cd Level Determination

Rapeseed seedlings were grown in the hydroponic system used by Tocquin et al. (2003) with slight modifications (Wan et al., 2017), in three different environments (E1: greenhouse, 2013; E2: greenhouse, 2014; E3: natural environment, 2014). The full-strength nutrient solution contained 0.5 mmol L^-1^ NH_4_H_2_PO_4_, 3 mmol L^-1^ KNO_3_, 2 mmol L^-1^ Ca(NO_3_)_2_, 1 mmol L^-1^ MgSO_4_, 0.09 mmol L^-1^ Na_2_EDTA (ethylenediamine-*N,N,N′,N′*-tetraacetic acid disodium), 0.09 mmol L^-1^ FeSO_4_, 22.5 μmol L^-1^ H_3_BO_3_, 10 μmol L^-1^ MnSO_4_, 0.10 μmol L^-1^ (NH_4_)_6_Mo_7_O_24_, 0.35 μmol L^-1^ ZnCl_2_, and 0.20 μmol L^-1^ CuSO_4_. The pH of the solution was adjusted to 6.7 with 1.0 mol L^-1^ NaOH. The seedings were cultivated in the greenhouse at 25°C under a 16 h photoperiod, and 2500 Lx illumination intensity, and at 20°C in the dark. The seedlings were germinated in an open space outdoors, but were covered with glass such that they received sunshine but were not negatively affected by rainwater.

Thirty healthy seeds from each rapeseed line of the 419 accessions were germinated over a 7-day period in a salver (60 cm × 40 cm × 10 cm) containing deionized water. A medical gauze sheet was placed on the surface of the salver, and it was separated into 96 plots using nylon filament. The seedings were planted in a hydroponic system after a 7-day germination period. A black plastic plate (60 cm × 40 cm × 10 cm) with 77 (7 × 11) holes (the diameter was 1.5 cm) was placed on the salver that contained Hoagland solution. A single seedling of each line was planted in one hole, and six salvers were used to cultivate the 419 lines, which were randomly arranged in the holes for a single replication. Five replications were performed with five similar seedlings from each line. Ten liters of 0.25× Hoagland solution was used as the hydroponic growing medium for 3 days, followed by 10 L of 0.5× Hoagland solution for the subsequent 3 days and 10 L of 1× Hoagland solution over the following 7 days. Then the seedlings were exposed to a 1× Hoagland nutrient solution containing 5 mg L^-1^ Cd^2+^ (CdCl_2_) for 3 weeks. Hoagland solution was renewed every 7 days throughout the experiment. After exposure to Cd^2+^ for 3 weeks, the roots were washed thrice with 5 mmol L^-1^ CaCl_2_ and excised from the shoots. The roots and shoots were separated, dried in an oven at 105°C for 30 min, dried at 80°C for 72 h to a constant weight, and then used for Cd determination. Root-to-shoot Cd translocation was calculated based on the ratio of shoot Cd content to the Cd content in the whole plant per the following equation:

$$CT=\frac{SCC*SDW}{SCC*SDW+RCC*RDW}$$

^∗^Trait: RCC, root Cd concentration; SCC, shoot Cd concentration; RDW, roots dry weight; SDW, shoots dry weight; CT, Cd translocation coefficient.

To determine the Cd^2+^ content, dried shoots or roots harvested from five replicates per line were pulverized using a 50 mL centrifuge tube containing a pair of 6 mm steel balls spun for 5 min in a high-speed oscillator (F&FM, Model SK450; Australia). Dried pulverized samples (DPSs) of ∼0.02 g were digested over 3 days in 2 mL 70% v/v nitric acid in a 14 mL centrifuge tube and at 100°C for 2 h exposures. The digests were diluted with ultrapure water to 12 mL volume for metal determination. The Cd concentrations in the digested solutions (CCDS) were determined with graphite furnace atomic absorption spectrometry [Model WFX-120A; Beijing Beifen Ruili Analytical Instrument (Group) Co. Ltd., Beijing, China]. Standard solution of Cd (GSB 04-1721-2004, 1000 μg mL^-1^, National Standard Material Center, China) were employed to draw standard curves and certified standard samples [GBW(E)100341, National Standard Material Center, China] were used to check determination accuracy. The shoot (root) Cd content was calculated as [CCDS of shoot (root) × 12 mL]/DPS.

### Statistical Analysis of Phenotypic Data

Phenotypic data, correlation, and significance were analyzed using SPSS v22 (IBM SPSS, Armonk, NY, United States). Differences at the acceptance levels *P* < 0.05 and *P* < 0.01 were considered significant and highly significant, respectively. Descriptive statistics of the traits and Pearson’s correlation coefficients were determined using the mean values of all phenotypic data of the 419 rapeseed accessions. The frequency distribution of each trait was obtained using R v. i386 3.3.3. The best linear unbiased prediction (BLUP) and broad-sense heritability for the traits was calculated by the R package lme4 (Merk et al., 2012). It was expressed as *h*^2^ = O’^2^*_g_*/(O’^2^*_g_* + O’^2^*_ge_*/*n* + O’^2^*_e_*/*nr*), where O’^2^*_g_* is the genetic variance, O’^2^*_ge_* is the variance of the interaction of the genotype with the environment, O’^2^*_e_* is the error variance, *n* is the number of environments, and *r* is the number of replicates (Wan et al., 2017). The BLUP values were employed as phenotypes for the association analysis (Piepho et al., 2008; Li et al., 2014).

### Genome-Wide Association Study and Candidate Gene Identification

The Brassica 60K Illumina^®^ Infinium SNP array (Xu et al., 2015) was used to evaluate the genotypes of the 419 rapeseed accessions. A total of 19,167 high-quality SNPs (Call Rate > 0.7; SNP Call Freq > 0.75; Minor Freq > 0.05; AA, BB frequency > 0.03; GenTrain Score > 0.5) were used for population structure and relative kinship analyses as described by Sun et al. (2016b).

The association analysis was conducted using TASSEL v. 4.0 (Bradbury et al., 2007) as well as four models: GLM, without considering Q or K; GLM, considering Q(Q); MLM, considering K(K); and MLM, considering Q and K(Q + K). The SNP markers significantly correlated with Cd accumulation were identified at a probability level of *P* ≤ 0.0001 or -log(*P*) ≥ 4.00, in accordance with the published literature (Wan et al., 2017). The *B. napus* genes orthologous to *A. thaliana* Cd-related genes located within 500 kb of the peak SNPs were identified based on the *B. napus* reference genome (Chalhoub et al., 2014). The genes that responded to the different Cd concentrations were regarded as candidate genes for Cd accumulation traits.

Two lines, L28 and L83 with the least and highest average root Cd concentrations, respectively, in three environments, were employed for qRT-PCR analysis. The plants were cultivated and exposed to Cd as described in Section “Hydroponic Experiments and Cd Level Determination.” The roots were separated from the seedlings (three biological replicates) after exposure to Cd for 24 h. The expression level of 10 candidate genes was analyzed. The qRT-PCR analysis was conducted according to previous methods (Zhang et al., 2017).

## Results

### Genotypic Variations in Cd Concentration and Translocation at the Seedling Stage

The 419 rapeseed accessions were cultivated in three environments (E1: greenhouse, 2013; E2: greenhouse, 2014; E3: natural environment, 2014) at the seedling stage. Shoot and root Cd concentrations and Cd translocation were compared after 3-week exposure to 5 mg L^-1^ Cd^2+^ in the hydroponic solutions. The *h*^2^ values for Cd concentration in shoot, Cd concentration in root, and Cd translocation were 85.3, 78.1, and 69.8%, respectively (**Table 1**). The results indicated that these Cd-related traits for a given genotype in the three environments are almost identical. The fact that the shoot Cd concentration was higher than the root Cd concentration implies that the former is less influenced by the environment than the latter. There were significantly positive correlations among the three hydroponic culture experiments for all three Cd traits: Pearson’s correlation coefficients ranged from 0.57 to 0.85 (**Table 2**).

**Table 1:** Phenotypic variation for Cd accumulation in 419 accessions of *Brassica napus.*

Trait	Environment	Minimum	Maximum	Mean	*SD*	CV (%)	Skewness	Kurtosis	*h*^2^ (%)
SCC	E1	106.2	337.39	210.67	43.01	20.42	0.09	-0.36	85.3
	E2	109	463.11	224.80	50.87	22.63	0.76	1.04	85.3
	E3	142.18	396.88	257.55	45.34	17.61	0.56	0.39	85.3
RCC	E1	728.57	3047.02	1690.86	394.18	23.31	0.46	0.46	78.1
	E2	642.79	3404.59	1697.46	507.83	29.92	0.66	0.33	78.1
	E3	767.53	3847.2	1882.24	489.14	25.99	0.55	0.62	78.1
CT	E1	0.19	0.57	0.37	0.08	20.55	0.05	-0.38	69.8
	E2	0.16	0.67	0.39	0.10	26.84	0.19	-0.61	69.8
	E3	0.14	0.65	0.40	0.08	20.00	-0.32	0.38	69.8


*Trait: RCC, root Cd concentration (μg g^-*1*^); SCC, shoot Cd concentration (μg g^-*1*^); CT, Cd translocation coefficient. RCC and SCC are expressed in dry weight (DW) basis. Environment: E1, greenhouse in 2013; E2, greenhouse in 2014; E3, natural environment in 2014. SD, standard deviation; CV (%), coefficient of variation; *h*^*2*^, heritability of mean values.*

**Table 2:** Phenotypic correlations between the Cd accumulation traits based on the trait mean values of plants grown in the three environments.

	SCC1	RCC1	CT1	SCC2	RCC2	CT2	SCC3	RCC3	CT3
SCC1	1	0.03	0.60^∗∗^	0.85^∗∗^	0.08	0.37^∗∗^	0.81^∗∗^	0.07	0.31^∗∗^
RCC1		1	-0.60^∗∗^	-0.00	0.84^∗∗^	-0.49^∗∗^	0.04	0.82^∗∗^	-0.53^∗∗^
CT1			1	0.54^∗∗^	-0.44^∗∗^	0.60^∗∗^	0.49^∗∗^	-4.44^∗∗^	0.62^∗∗^
SCC2				1	0.05	0.49^∗∗^	0.77^∗∗^	0.01	0.31^∗∗^
RCC2					1	-0.57^∗∗^	0.09	0.82^∗∗^	-0.51^∗∗^
CT2						1	0.33^∗∗^	-0.49^∗∗^	0.53^∗∗^
SCC3							1	0.07	0.40^∗∗^
RCC3								1	-0.64^∗∗^
CT3									1


*Trait: SCC1, shoot Cd concentration in E1; RCC1, root Cd concentration in E1; CT1, Cd translocation coefficient in E1; SCC2, shoot Cd concentration in E2; RCC2, root Cd concentration in E2; CT2, Cd translocation coefficient in E2; SCC3, shoot Cd concentration in E3; RCC3, root Cd concentration in E3; CT3, Cd translocation coefficient in E3. ^∗^*P* < 0.05, ^∗∗^*P* < 0.01.*

**FIGURE 1:**
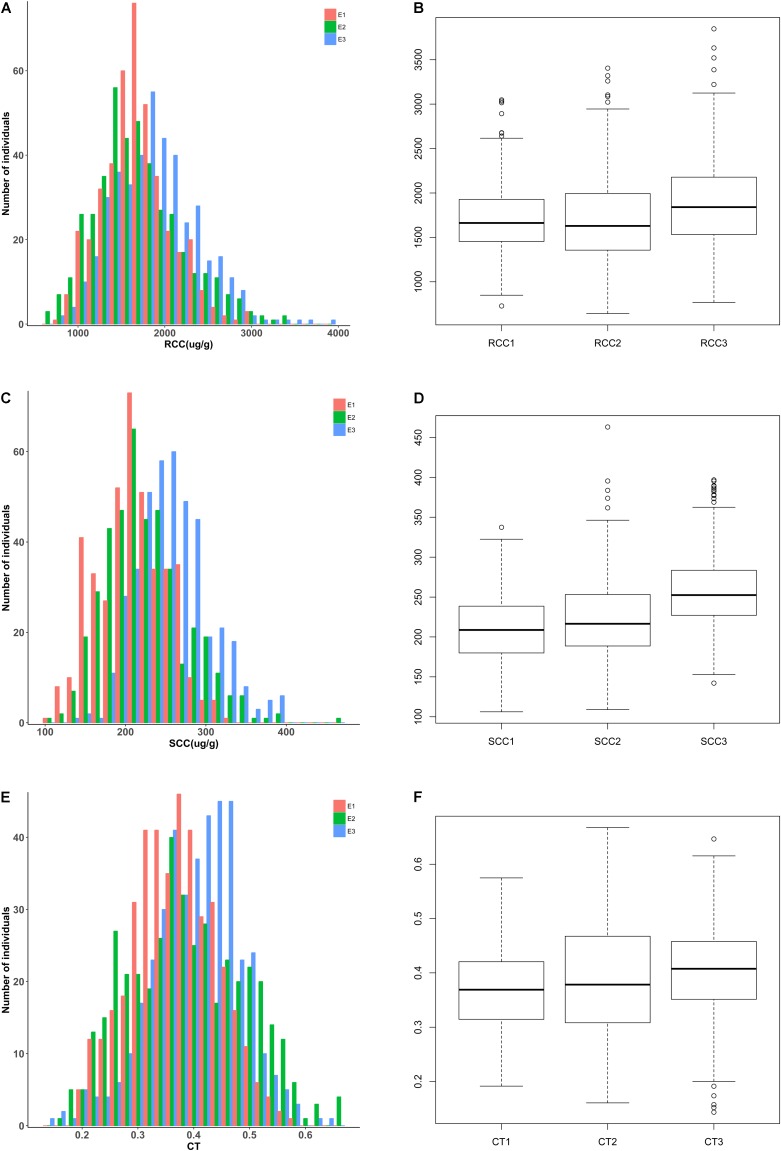
Genetic variation for Cd accumulation in root, shoot, and root/shoot translocation in the association panel of 419 accessions grown in three environments. **(A,B)** Root Cd concentration; **(C,D)** shoot Cd concentration; **(E,F)** Cd translocation coefficient. SCC1, shoot Cd concentration in E1; RCC1, root Cd concentration in E1; CT1, Cd translocation coefficient in E1; SCC2, shoot Cd concentration in E2; RCC2, root Cd concentration in E2; CT2, Cd translocation coefficient in E2; SCC3, shoot Cd concentration in E3; RCC3, root Cd concentration in E3; CT3, Cd translocation coefficient in E3. E1, greenhouse in 2013; E2, greenhouse in 2014; E3, natural environment condition in 2014.

There were large genotypic variations in the Cd concentration and translocation across the three environments. The root Cd concentrations (RCC) ranged from 728.57 to 3047.02 μg g^-1^ dry weight (DW) in E1, 642.79 to 3404.59 μg g^-1^ DW in E2, and 767.53 to 3847.20 μg g^-1^ DW in E3 (**Figures 1A,B** and **Table 1**). The average root Cd concentration ranged from 1690.86 ± 394.18 to 1882.24 ± 489.14 μg g^-1^ DW (**Table 1**). Two accessions each had among the 10 lowest [L28 (SWU101) and L87 (97096)] and 10 highest [L83 (07022) and L482 (Shengli)] root Cd concentrations in all three environments (Supplementary Data S1 and Supplementary Table S4). The Cd concentration in shoots (SCC) ranged from 106.2 to 337.39 μg g^-1^ DW in E1, 109 to 463.11 μg g^-1^ DW in E2, and 142.18 to 396.88 μg g^-1^ DW in E3 (**Figures 1C,D** and **Table 1**). The average shoot Cd concentration ranged from 210.67 ± 43.01 to 257.55 ± 45.34 μg g^-1^ DW (**Table 1**). Two accessions [L62 (10-1358) and L87 (97096)] had among the 10 lowest shoot Cd concentrations, and three accessions [L362 (SWU100), L397 (Zheshuang8), and L525 (Chuxianbaihua)] had among the 10 highest shoot Cd concentrations in all three environments (Supplementary Data S1 and Supplementary Table S4). The coefficient of Cd translocation (CT) varied from 0.19 to 0.57 in E1, 0.16 to 0.67 in E2, and 0.14 to 0.65 in E3 (**Figures 1E,F** and **Table 1**). The average Cd translocation coefficient ranged from 0.37 ± 0.08 to 0.40 ± 0.08 (**Table 1**). One accession [L149 (Ningyou1)] had among the 10 lowest Cd translocation coefficients, but none of the accessions had any of the 10 highest Cd translocation coefficients in any of the three environments (Supplementary Data S1 and Supplementary Table S4). Unimodal distribution patterns were observed for all traits among these accessions in the three independent environments (**Figures 1A,C,E**).

Pearson’s correlation coefficients were calculated among the three Cd traits across environments. Significant positive correlations (*P* < 0.01) were identified between Cd concentration in roots and Cd translocation, and significant negative correlations (*P* < 0.01) were identified between Cd concentration in shoots and Cd translocation of the three environments (**Table 2**). However, no significant correlations were noted between the shoot and root Cd concentrations (**Table 2**).

### Determining the Most Appropriate Models

**FIGURE 2:**
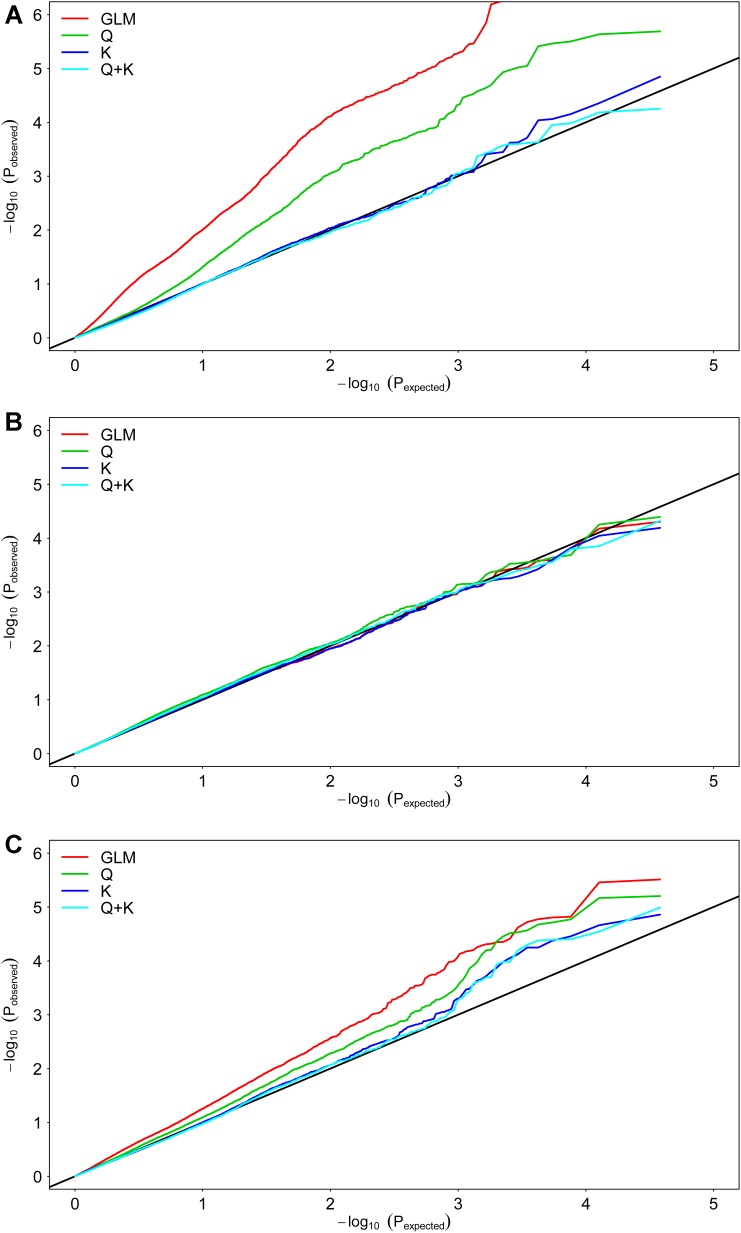
Quantile–quantile plots of estimated –log10(*P*) from association analysis of Cd accumulation traits in rapeseed accessions. **(A)** Root Cd concentration (RCC). **(B)** Shoot Cd concentration (SCC). **(C)** Cd translocation coefficient (CT). GLM considering no Q and K; Q, GLM considering Q; K, MLM considering K; Q + K, MLM considering Q and K;

We selected 19,167 SNPs distributed across the entire genome to analyze population structure and genetic kinship. The 419 genotypes were divided into two subgroups, designated P1 (51 accessions) and P2 (325 accessions), and 43 accessions mixed (Supplementary Table S1). The Q matrix and the relative kinship coefficients (K) were analyzed in a previous study (Sun et al., 2016b). To detect the genetic factors associated with Cd concentration and translocation, we performed a GWAS using the Cd concentration and translocation BLUP data from the three environments (Supplementary Data S2). To determine the most suitable model for GWAS, the fit was tested with the three traits (**Figure 2**). The best-fit model changed with rapeseed traits, based on quantile–quantile plots. For example, the K and Q + K models adapted best to the expected *P*-values for RCC (**Figure 2A**). All four models adapted well for the expected *P*-values of SCC (**Figure 2B**). The Q, K, and Q + K models fit best to the expected *P*-values for CT (**Figure 2C**). The Q model was applied to the GWAS to use the genotypic and phenotypic data of this study most efficiently. The Q + K model was used for association mapping to control for false positives.

### GWAS Analysis for Cd Concentration and Translocation

**FIGURE 3:**
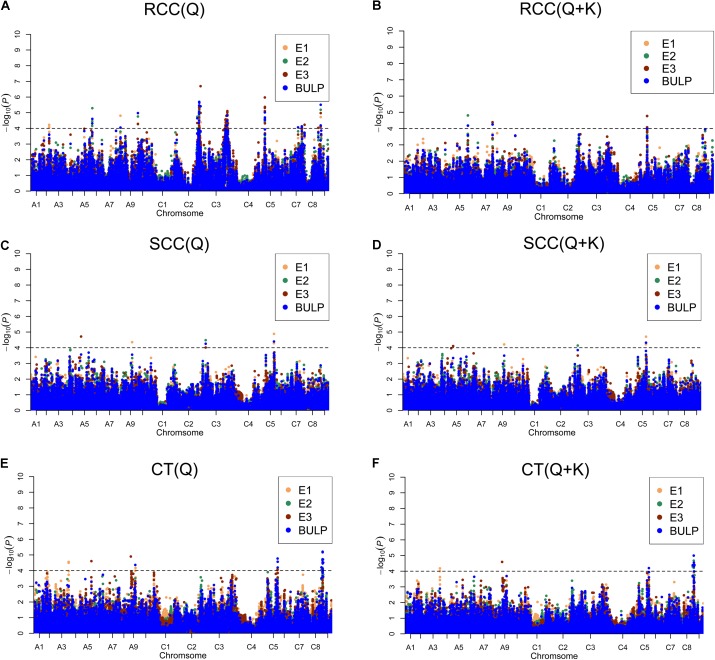
Manhattan plots generated from genome-wide association analysis results for Cd accumulation traits using Q and Q + K models. **(A)** RCC (Q). **(B)** RCC (Q + K). **(C)** SCC (Q). **(D)** SCC (Q + K). **(E)** CT (Q). **(F)** CT (Q + K). E1, greenhouse in 2013; E2, greenhouse in 2014; E3, natural environment condition in 2014; BLUP, best linear unbiased prediction values. Gray horizontal line depicts significantly threshold (*P* < 0.0001).

We identified 98 significantly [-log(*P*) > 4.0 or *P* < 0.0001] associated SNPs of shoot and root Cd concentration and Cd translocation using the two aforementioned models (Supplementary Table S2). Among the 98 associated SNPs, 20 were identified in E1, 26 in E2, and 68 in E3. Forty-four SNPs were identified using BLUP values in the three environments (**Figure 3** and Supplementary Table S2). Additionally, 32.7% (32/98) of the SNPs were verified in more than one environment (including BLUP), which indicates high reliability. For shoot and root Cd concentration and Cd translocation, 6, 72, and 21 SNPs were detected, respectively (**Figure 3** and Supplementary Table S2). Bn-A07-p21371577 on chromosome A07 was detected by the Q + K model but not the Q model for root Cd concentration in both E3 and BLUP. Bn-scaff_23186_1-p340266 on chromosome C05 was found for both shoot Cd concentration and Cd translocation, meaning that the SNP genetically contributed to both these traits. Five SNPs for root Cd concentration were validated in all environments and with BLUP (Supplementary Table S2).

To integrate putative QTLs, we employed a previously described method (Liu et al., 2016) with a slight modification. The SNPs were used as identifiers for the same QTL if the lead and following SNPs were within 500 kb of each other or if the pairwise *r*^2^ LD estimator between the lead and the following SNPs was >0.35. In all, the 98 significant SNPs constituted 35 QTLs and explained 3.49–7.57% of the phenotypic variation in oilseed varieties (**Table 3** and Supplementary Table S2). The six significantly associated SNPs constituted five QTLs on chromosomes A5, A9, C3, and C5 for shoot Cd concentration and accounted for 3.61–5.03% of the phenotypic variation (**Table 3** and Supplementary Table S2). One of these QTLs was validated in more than one growth environment. For root Cd concentration, 72 significantly associated SNPs, forming 20 QTLs, were located on 11 chromosomes and explained 3.49–7.57% of the phenotypic variation (**Table 3** and Supplementary Table S2). Among those QTLs, seven were identified in more than one growth environment. The SNPs Bn-A10-p1606279 and Bn-scaff_17190_1-p9206 were detected in all three environments and with BLUP (**Table 3**). The 21 significantly associated SNPs constituted 10 QTLs on chromosomes A3, A5, A9, C5, and C8 for Cd translocation and explained 3.74–5.53% of the phenotypic variation (**Table 3** and Supplementary Table S2). Three of these stable QTLs were validated in more than one growth environment to illustrate their impact on phenotype.

**Table 3:** SNPs significantly associated with Cd accumulation and translocation in the three growth environments and BLUP.

	SNPs^a^	Chr	Site(b)	Allele	MAF	Q		Q + K		Env^c^
								
						-Log10P	*R*^2^ (%)^b^	-Log10P	*R*^2^ (%)^b^	
CT	Bn-A03-p28233701	A03	26700046	A/G	0.212	4.50	4.63	4.00	4.24	E1
	Bn-A05-p21037865	A05	19196309	A/G	0.464	4.60	5.53			E3
	Bn-A09-p17415894	A09	13189315	A/G	0.261	4.90	5.23	4.59	5.08	E3
	Bn-A05-p15670035	A09	22929483	A/C	0.084	4.36	3.94			BLUP
	Bn-A09-p25633407	A09	23746951	A/C	0.284	4.17	3.77			E1
	Bn-scaff_20270_1-p1323200	C05	41750625	A/C	0.481	4.14	3.74			E1
	Bn-scaff_20376_1-p361323	C05	42267126	A/G	0.277	4.05	4.67			E3
		C05	42267126			4.77	5.55			BLUP
	Bn-scaff_16445_1-p728844	C08	36063670	A/G	0.267			4.29	4.61	E1
		C08	36063670	A/G	0.267			4.67	5.18	E2
		C08	36063670	A/G	0.267			4.99	5.55	BLUP
	Bn-scaff_16445_1-p584652	C08	36224948	A/G	0.453	4.02	4.94			E1
		C08	36224948			4.05	4.98			E3
		C08	36224948			5.17	6.48			BLUP
	Bn-scaff_16445_1-p268218	C08	36557402	A/G	0.401	4.01	5.39			E2

RCC	Bn-A02-p23708516	A02	21917889	A/G	0.220	4.22	4.56			E1
	Bn-A06-p3013547	A06	2901679	A/G	0.452	5.28	5.94	4.81	5.68	E2
		A06	2901679			4.60	5.03	4.18	4.81	BLUP
	Bn-A07-p21371577	A07	22921984	A/G	0.171			4.38	4.49	E3
		A07	22921984	A/G	0.171			4.25	4.34	BLUP
	Bn-A08-p11452221	A08	9295219	A/C	0.273	4.81	4.28			E1
		A08	9295219			4.05	3.52			BLUP
	Bn-A08-p13009633	A08	10785697	A/C	0.239	4.05	3.49			E3
	Bn-A10-p1606279	A10	2098086	A/T	0.110	4.30	4.06			E1
		A10	2098086			4.76	4.59			E2
		A10	2098086			4.27	3.98			E3
		A10	2098086			4.97	4.75			BLUP
	Bn-scaff_15712_9-p621118	C02	39348154	A/G	0.490	4.34	4.48			E3
	Bn-scaff_15918_1-p318193	C02	42242763	A/G	0.347	5.37	6.05			E2
		C02	42242763			5.35	5.84			E3
		C02	42242763			5.69	6.33			BLUP
	Bn-scaff_15918_1-p318465	C02	42243137	A/G	0.346	4.63	5.06			E1
	Bn-scaff_26086_1-p11779	C02	44930131	A/G	0.274	6.69	7.57			E3
	Bn-scaff_17042_1-p511184	C03	53628383	T/C	0.264	4.46	4.57			BLUP
	Bn-scaff_17042_1-p516850	C03	53639315	A/G	0.316	5.10	5.35			E3
	Bn-scaff_17042_1-p517224	C03	53639589	A/C	0.273	4.22	3.75			E2
	Bn-scaff_16888_1-p1786111	C04	45922939	A/G	0.471	4.41	5.28			E2
	Bn-scaff_16888_1-p1786897	C04	45923726	A/G	0.483	5.97	6.67	4.77	5.50	E3
		C04	45923726			5.02	5.56			BLUP
	Bn-scaff_17291_1-p239559	C07	33143985	A/G	0.342	4.06	4.95			BLUP
	Bn-scaff_16069_1-p3375579	C07	39782757	A/G	0.442	4.02	4.44			E2
	Bn-scaff_16110_1-p685258	C07	44204542	A/G	0.456	4.23	4.79			E3
	Bn-scaff_16445_1-p2635783	C08	34269830	A/G	0.284	4.03	4.09			E3
	Bn-scaff_17190_1-p9206	C09	3592360	A/G	0.216	4.72	5.10			E1
		C09	3592360			5.19	5.75			E2
		C09	3592360			4.98	5.37			E3
		C09	3592360			5.50	6.04			BLUP

SCC	Bn-A05-p4569670	A05	4422400	A/C	0.084	4.71	4.33	4.11	3.84	E3
	Bn-A09-p23928124	A09	22308543	A/C	0.368	4.35	3.96	4.21	3.96	E1
	Bn-scaff_18322_1-p2327100	C03	6887429	A/C	0.189	4.48	4.09	4.14	3.88	E2
		C03	6887429			4.01	3.61			E3
		C03	6887429			4.25	3.86			BLUP
	Bn-scaff_23186_1-p340708	C05	41119696	A/G	0.405	4.29	5.03	4.22	5.03	E1
	Bn-scaff_23186_1-p340266	C05	41120129	A/C	0.383	4.39	4.00	4.32	4.08	BLUP


*^*a*^Only the lead SNPs at a defined locus are shown. ^*b*^Percentage of phenotypic variance explained by the lead SNP. ^*c*^E1 greenhouse in 2013, E2 greenhouse in 2014, E3 natural environment in 2014, BLUP: best linear unbiased prediction values.*

**FIGURE 4:**
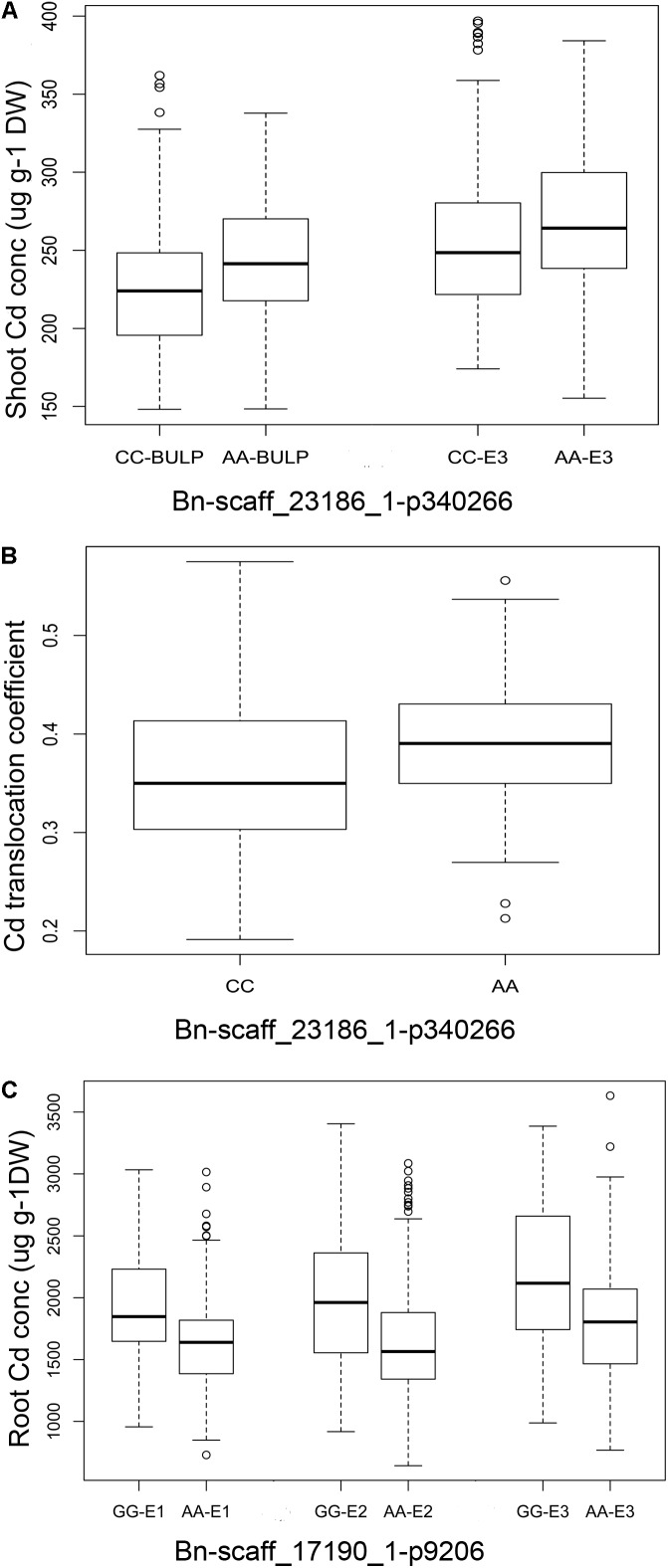
Phenotypic differences between lines with different alleles of the SNPs associated with Cd accumulation traits in three growth environments. **(A)** *Bn-scaff_23186_1-p340266* associated with root Cd concentration in E1 (*P* < 0.001) and BLUP (*P* < 0.0001). **(B)** *Bn-scaff_23186_1-p340266* associated with Cd translocation coefficient in E1 (*P* < 0.0001). **(C)** *Bn-scaff_17190_1-p9206* associated with root Cd concentration in three environments (all *P* < 0.0001).

Two QTLs were used to illustrate the impact of these stable QTLs on phenotype. The QTL on chromosome C5 (Bn-scaff_23186_1-p340266) was identified with both shoot Cd concentration (E1, BLUP) and Cd translocation. Bn-scaff_17190_1-p9206 on chromosome A09 was associated with root Cd concentration. The polymorphism of the markers fit well with the variation of the phenotypic data, and the average Cd concentrations in shoot of the CC genotype were 206.35 μg g^-1^ DW (E1) and 226.47 μg g^-1^ DW (BLUP). In the AA genotype, they were 219.41 μg g^-1^ DW (E1) and 243.55 μg g^-1^ DW (BLUP). The average Cd translocation was 0.36 in the CC genotype and 0.39 in the AA genotype (Supplementary Data S1 and **Figures 4A,B**). The Cd translocation and shoot Cd concentrations were significantly higher (*P* < 0.001) in the AA phenotype than those in the CC phenotype (**Figures 4A,B**). The root Cd concentrations in the accessions carrying the GG allele of Bn-scaff_17190_1-p9206 were significantly higher (*P* < 0.001) than those with the AA allele in all three environments (**Figure 4C**).

### Comparison of QTL Positions for Candidate Genes of Cd Accumulation and RT-PCR Verification

**FIGURE 5:**
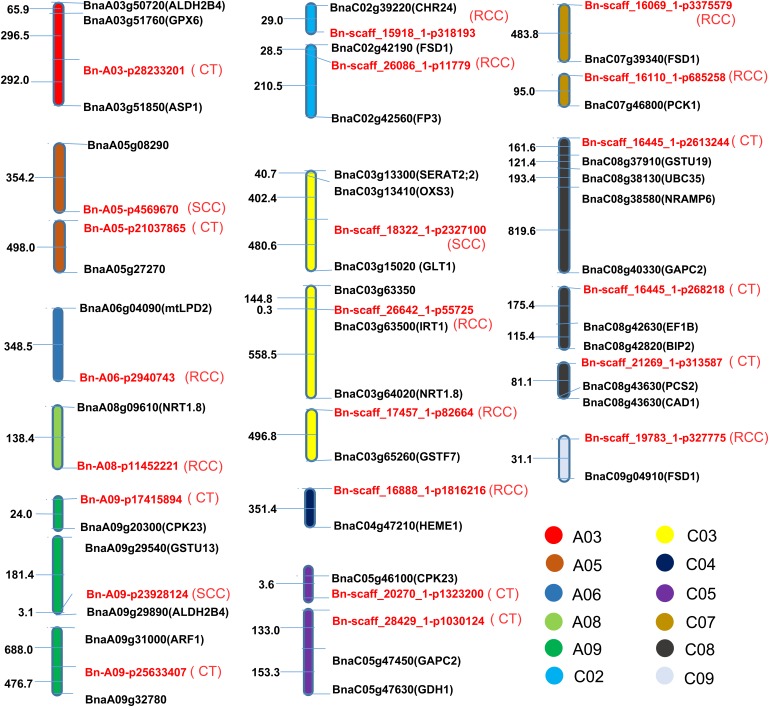
The distribution pattern of candidate genes and the corresponding SNPs associated with Cd accumulation. The abbreviations of orthologous genes in *Arabidopsis thaliana* are shown in brackets after the candidate genes. SNPs are marked in red. Numbers represent the relative distances in the genome, 1 = 1 kb.

Several genes affecting Cd accumulation have been identified in *A. thaliana* and rice. We identified Cd-related orthologous genes of *A. thaliana* in *B. napus* located within 500 kb of each SNP. We obtained 32 candidate Cd accumulation-related genes (**Figure 5** and Supplementary Table S3) following comparison to the *B. napus* reference genome (Chalhoub et al., 2014). The physical distance between the significant SNPs ranged from 0.33 to 497.97 kb (**Figure 5**). Totally, 7, 10, and 15 genes were identified for shoot Cd concentration, root Cd concentration, and Cd translocation, respectively. Four genes had multiple copies on different chromosomes. In particular, the *A. thaliana* ortholog ALDH2B4 (AT3G48000) was identified on chromosomes A3 and A9 for root Cd concentration and shoot Cd concentration, respectively. The phytochelatin synthase (PCS) orthologs cadmium-sensitive 1 (CAD1) and phytochelatin synthase 2 (PCS2) were defined at the same site on chromosome C08 at 81.08 kb from the SNP Bn-scaff_21269_1-p313587 for Cd translocation. Comparison between the significant SNPs detected in this study and the gene loci in the *A. thaliana* genome indicated that *B. napus* had orthologous genes to NRAMP6, IRT1 (iron-regulated transporter 1), CAD1, PCS2, and others from *Arabidopsis* (Supplementary Table S3). Four genes were found to have more than one orthologous gene copy in different chromosomes associated with the same trait. For example, three FSD1 orthologs located on chromosomes C02, C04, and C07 were found to be associated with root Cd concentration (Supplementary Table S3). Finally, two ALDH2B4 orthologs located on chromosomes A03 and A09 were associated with root Cd concentration and Cd translocation, respectively.

**FIGURE 6:**
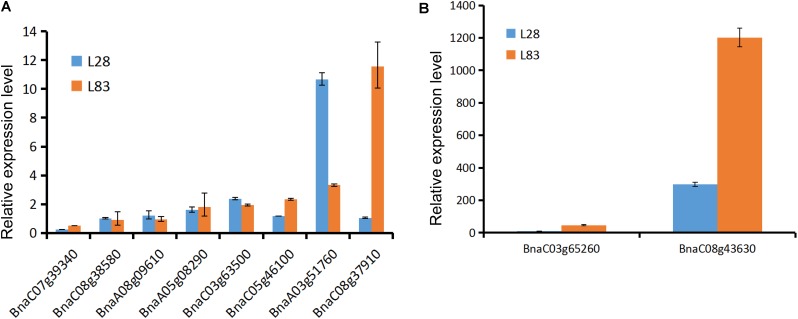
The genes expression level in two lines after exposure to Cd based on qRT-PCR. The *y*-axis represents Cd-induced expression fold change relative to control treatment. **(A)** The genes expression level less than 15 times in both lines. **(B)** The genes expression level more than 15 times in one or both lines.

We determined the expression level of 10 candidate genes in the roots of two lines with considerable differences in Cd concentrations in the roots (L28 with low root Cd concentrations and L83 with high root Cd concentrations) using qRT-PCR analysis. The primers were described in **Table 4**. The result showed that the expression level of BnaC03g65260, BnaA03g51760, and BnaC08g43630 was significantly higher after exposure to Cd than in the CK in both lines. BnaC08g37910 expression levels were significantly higher in L83 but no differences were noted in L28. In contrast, the expression level of BnaA03g51760 was three times higher in L28 than in L83 (**Figure 6**).

**Table 4:** Primers for real-time quantitative PCR.

Gene name	Left primer	Right primer
Actin	GGAAGCTCCTGGAATCCATGAGA	TCTTTGCTCATACGGTCAGCAATTCC
BnaA05g08290	TTCCTGCTTTGGATCTCATC	TTCAGTCTGAAGAATGTCGG
BnaA08g09610	TTGAGTTCTTCAACAGCCAC	ATCTCAACAAAGGGCACTTGGA
BnaC03g63500	TAGCATCAAATTTCAGATCAAGTGCTTTGC	TTAAGCCCACTTGGCGACCACC
BnaC03g65260	GTGCAGCCATTTGGTCAAG	TTATGACGATTTAATTTCTCTGATGAGAG
BnaC07g39340	TGTGAATCCCCTTGTGCTTG	TTAAGCAGAAGAGGAAGAAGC
BnaA03g51760	GGGGTTTCCTTGAAATCTTGG	GTTAAACCACATTTTGAAGCAACGT
BnaC05g46100	CTACTGATCTCCAACAGCTTTCT	ACAGATATCTCCTCGCTCTTC
BnaC08g38580	TGAGATTCTGGCTAAGTTATTCC	TTCTTGAGATGTTGAAGGAGGA
BnaC08g43630	GAAGACAGTCAGTCATATGTTCC	CGGACGGGTAAGTCTTC
BnaC08g37910	ACATAGCCCTGATTGGATTC	CTACCCGACCCGTTTTTTC


## Discussion

### Cd Exposure and Phenotypic Variation

Cd accumulation in rapeseed is a complex quantitative trait. Meanwhile, as this plant has a large genome and relatively few known mutations, little has been reported to date with respect to genotypic differences in Cd accumulation. Moreover, the rapeseed genes involved in Cd accumulation have not yet been reported. Identification of the accessions with low or high Cd accumulation would be useful for both breeding purposes and research on the genes involved in the trait. In the present study, we evaluated rapeseed shoot and root Cd concentration and Cd translocation by exposing seedlings to 5 mg L^-1^ Cd^2+^ in hydroponic culture for 3 weeks. Wide genotypic differences in Cd accumulation were found (**Figure 1**), as has been observed in other species such as rice (Ueno et al., 2009), *A. thaliana* (Chao et al., 2012), and barley (Wu D. et al., 2015). Furthermore, these genotypic variations were present in both the roots and shoots and in both greenhouse- and field-raised plants (**Figure 1**). These results suggest that Cd concentration and translocation are genetically controlled in rapeseed. Compared with previous rapeseed studies (Wu Z. et al., 2015), this experiment identified a wider range of genotypic differences in Cd accumulation (**Figure 1**). One possible explanation for this discrepancy might be that we used 419 accessions originating from 10 countries in four continents. In contrast, earlier studies only tested a few accessions.

Our results suggest that the root and shoot might accumulate Cd by different mechanisms. Shoot Cd concentrations and Cd translocation were significantly and positively correlated (*P* < 0.01). There was also a significant negative correlation between Cd translocation and root Cd concentration. Furthermore, the shoot and root Cd concentrations were not significantly correlated. These findings contradict those reported for barley (Wu D. et al., 2015), possibly because the rapeseed plants in the present study were exposed to higher Cd dosages than were the barley plants in that study. We obtained relatively higher Cd translocation coefficients for rapeseed than those reported for barley (Wu D. et al., 2015) but similar to those determined for Japanese honeysuckle (*Lonicera japonica* Thunb.) at higher Cd concentrations (Liu et al., 2009). We also obtained relatively higher Cd translocation coefficients for rapeseed than previously reported (Wu Z. et al., 2015); this may because we used higher Cd concentrations and more germplasm.

Overall, the roots and shoots of the rapeseed accessions tested in this study accumulated significantly more Cd and at lower doses and exposure times than other plant species tested in the past. The maximum rapeseed shoot and root Cd concentrations determined in this study were >450 μg g^-1^ DW and >3800 μg g^-1^ DW, respectively, after cultivated in 5 mg L^-1^ Cd^2+^ for 21 days (**Figure 1A**, **Table 1**, and Supplementary Data S1). In contrast, the shoot Cd concentration in *Iris lactea* var. *chinensis* was only 529 μg g^-1^ DW after cultivation in 80 mg L^-1^ Cd^2+^, and the shoots of *Iris tectorum* accumulated only 232 μg g^-1^ DW Cd after cultivation in 40 mg L^-1^ Cd^2+^ for 42 days (Han et al., 2007). The roots and shoots of *Amaranthus hybridus* had Cd concentrations of 587.03 μg g^-1^ DW and 354.56 μg g^-1^ DW, respectively, after cultivation in 40 mg L^-1^ Cd^2+^ for 30 days (Zhang et al., 2010). *A. halleri* shoots accumulated Cd in the range of 569–1372 μg g^-1^ DW after cultivation in 5 μmol L^-1^ CdSO_4_ for 21 days (Meyer et al., 2015). Considering the large rapeseed biomass production and the value of this crop as a source of oil, Cd hyperaccumulator accessions might be better suited for phytoremediation than other plant species, and accessions with higher Cd accumulation would be more efficient than the varieties employed by earlier studies (Cojocaru et al., 2016).

Hyperaccumulator plants have several useful traits, such as high metal tolerance, the ability to concentrate metals in the shoots, and large metal transport coefficients (root-to-shoot translocation; Vamerali et al., 2010). High-yielding rapeseed with high metal contamination tolerance has great potential as a Cd hyperaccumulator (Cojocaru et al., 2016). The 5 mg L^-1^ Cd^2+^ treatment we used in this study to investigate genotypic Cd accumulation differences was similar to the doses tested in earlier phytoremediation experiments on *Iris* (Han et al., 2007), *A. hybridus* (Zhang et al., 2010), and *A. halleri* (Meyer et al., 2015). Therefore, the genotypic variations in Cd accumulation observed in the present study might be indicative of Cd distribution in other hyperaccumulator plant species as well, and rapeseed could be recommended for use in phytoremediation. In addition, accessions with relatively poorer Cd accumulation would fit for breeding for use as vegetable and oilseed crops.

### GWAS for Cd Accumulation Traits

In the present study, two models (Q and Q + K) were used to analyze the genetic architecture of Cd accumulation. There were many loci common to both models (Supplementary Table S2), but the methods differed in power and applicability. The Q test proved to be more robust than the Q + K test because the latter involved kinship, which reduced *P*-value inflation and generated false negative values (**Figure 3**). For example, Bn-scaff_15918_1-p318193 and Bn-scaff_26086_1-p11779 failed to reach the significance level in the Q + K model but not in the Q model even though their *Arabidopsis* orthologs CHR24, FSD1, and FP3 all participated in Cd disposition after the plants were exposed in all three environments. This finding was also corroborated by the BLUP value (Supplementary Table S3).

We detected 5, 20, and 10 QTLs for shoot and root Cd accumulation and Cd translocation, respectively, in various regions from the genome of *B. napus* (**Table 3**). Eleven of these 35 QTLs (31.4%) were detected in more than one environment and 68.6% (24/35) in a single environment. The results demonstrate that there were significant interactions between the growth environments and the QTLs. Most of the QTLs are organ-specific. For example, the QTLs associated with root Cd accumulation are not related to those detected for shoot Cd accumulation (**Tables 2**, **3**). Therefore, different genes affect Cd accumulation in different organs. Nevertheless, Bn-scaff_23186_1-p340266 was associated with shoot Cd concentration and Cd translation; therefore, the same genes might be involved in both processes. In addition, a strong positive correlation was detected between shoot Cd concentration and Cd translocation. The results of this study might therefore provide a reference for the genetic mechanism of rapeseed Cd accumulation.

### Cd Accumulation-Related Candidate Genes

Previous reports have identified the molecular mechanisms of Cd accumulation in rice (Sasaki et al., 2012), *Arabidopsis* (Wong and Cobbett, 2009; Chen et al., 2015), and *S. plumbizincicola* (Liu et al., 2017). In contrast, little has been reported about the genes involved in Cd accumulation in *B. napus*. The candidate genes detected in our study can be divided into several functional groups, including transcription factors, transporters, PCS, and enzymes. Orthologs for most of these candidate genes have been reported in *Arabidopsis* and other plant species.

The first step in iron accumulation in plants is transportation from soil to root. IRT1 is an important root transporter responsible for plant iron absorption from the soil. It is also the main portal or gateway for potentially toxic metals in plants (Barberon et al., 2014). IRT1 is also a broad-spectrum transporter that participates in the absorption of other divalent cations (Lux et al., 2011). When *A. thaliana* was treated with gibberellin in the presence of Cd^2+^, Cd accumulation was significantly reduced in the roots, and the upregulation of IRT1 expression was suppressed. On the other hand, the mitigation of Cd toxicity by gibberellin was not observed in the IRT1 knockout mutant irt1; hence, IRT1 might be involved in Cd^2+^ absorption (Zhu et al., 2012). Previous research has demonstrated that loss of IRT1 function in irt1 mutants decreases Cd levels significantly in the tissues of plants cultivated in the presence of Cd. Therefore, IRT1 might be responsible for root Cd absorbing from the growth environments (Vert et al., 2002). In this study, we identified a QTL (Bn-scaff_21386_1-p17474) on chromosome C03. It is only 0.33 kb from the *Arabidopsis IRT1* homolog associated with root Cd accumulation. Therefore, the *IRT1* homolog in rapeseed might have participated in Cd uptake from the nutrient solution.

Shoot Cd accumulation is controlled by root absorption, sequestration, xylem translocation, and other factors (Ueno et al., 2009; Clemens et al., 2013). NRAMP is another family of metal transporters potentially involved in Cd mobilization. Transgenic *Arabidopsis* plants overexpressing *AtNRAMP6* were hypersensitive to Cd even though the metal content in the plants remained unchanged. Conversely, an *AtNRAMP6* null allele could tolerate higher Cd toxicity than the wild-type. Modifications to AtNRAMP6, which functions as an intracellular metal transport factor, may affect Cd distribution/availability in the cell (Cailliatte et al., 2009). In the present study, Bn-scaff_16445_1-p2613244, a QTL for Cd translocation located on chromosome C08, was 476.38 kb away from NRAMP6. It accounted for 4.73% of the phenotypic variation (**Table 3** and Supplementary Table S3). NRAMP6 may, therefore, play a role in Cd translocation. Functional NRAMP6 characterization is required to verify whether it is a candidate gene of the QTL (Bn-scaff_16445_1-p2613244) detected on chromosome C08.

Cd^2+^ may bind to important cell components and biological macromolecules and damage plant cells after being transported into the root. Glutathione may detoxify Cd by preventing these covalent bonds from forming. In plants, GSTs detoxify heterocyclic xenobiotics by covalently binding glutathione (GSH) to the substrate and forming a glutathione-*S*-conjugate (Ishikawa, 1992). The *Bz2* gene of maize encodes a GST, the expression of which is significantly induced by heavy metals like Cd (Marrs and Walbot, 1997). In rice, the promoter separated from two tau-class GTS (GSTU) genes, OsGSTU5 and OsGSTU37, exhibited low background expression under normal conditions, but its expression was significantly induced by Cd exposure. Therefore, OsGSTU5 and OsGSTU37 could be used to improve plant heavy-metal tolerance (Qiu et al., 2015). We split out five GST family genes ranging from 5.89 to 496.78 kb distant from their respective significant Cd accumulation signals (**Table 3** and Supplementary Table S3). We also identified a QTL (Bn-A03-p28233201) 296.53 kb away from the glutathione peroxidase (GPX6) Cd translocation homolog in *Arabidopsis.* GST family homologs in rapeseed might have key roles in Cd accumulation.

The synthesis of phytochelatins (PCS) is one of the most important detoxification mechanisms for metal in plants (Cobbett and Goldsbrough, 2002). PCS have the general formula (gGlu-Cys)-*n*Gly (where *n* = 2–11). Upon As and/or Cd exposure, tobacco seedlings overexpressing *AtPCS1* exhibited much higher PC levels, root As and Cd accumulation, and detoxification capacity than those of wild-type plants (Zanella et al., 2015). The overexpression of *AtPCS1* in *Arabidopsis* increased both PC production and Cd detoxification ability if the metal was provided in specific concentrations (Brunetti et al., 2011). Compared to wild-type tobacco, transgenic plants expressing the PCS gene from hornwort (*Ceratophyllum demersum* L. accessions; CdPCS1) showed several-fold increases in PC content, precursor non-protein thiols, and As and Cd accumulation without significant decreases in plant growth (Shukla et al., 2012). *Saccharomyces cerevisiae* (yeast) cells exhibited higher As tolerance and accumulation after being transformed with OsPCS2a than did untransformed controls. In transgenic *Arabidopsis*, the overexpression of AtPCS2 resulted in constitutive PC accumulation, which was weaker than that of AtPCS1 and was not tissue-specific (Lee and Kang, 2005). In a liquid medium seedling assay, AtPCS2 partially amended the Cd hypersensitivity of an AtPCS1-deficient cad1-3 mutant. Constitutive AtPCS2-dependent PC synthesis suggests that AtPCS2 might have a physiological role other than metal detoxification (Kühnlenz et al., 2014). In the present study, using Q and Q + K models, we identified a QTL (Bn-scaff_21269_1-p313587) on chromosome C08 located 81.08 kb from the two *Arabidopsis* PCS homologs PCS1 and PCS2 associated with Cd translocation. Both homologs were at the same position. These findings indicate that PCS homologs may participate in Cd detoxification and accumulation, and should be a focus of future research on plant Cd accumulation.

The qRT-PCR analysis of 10 candidate genes in roots of two lines confirmed that the expression levels of four genes were increased significantly after exposure to Cd. Interestingly, these genes are homologous to the *Arabidopsis* genes GSTF7, GPX6, GSTU19, and PCS2, which are important elements for detoxification in roots. The results showed that the candidate genes were analyzed by GWAS effectively.

## Conclusion

We found a wide range of genetically controlled variation for Cd accumulation in rapeseed. Rapeseed might be better suited for phytoremediation than other tested plant species such as *Iris* and *A. hybridus* because it can accumulate and tolerate more Cd and suffers less growth inhibition than the other species. GWAS detected several QTLs of Cd accumulation in the roots and shoots, as well as for Cd translocation. Candidate genes were identified as homologs of *Arabidopsis* IRT1, NRAMP6, PCS1, PCS2, and GSTs, and could be cloned in future studies with a view toward genetically introducing Cd tolerance in other plant species.

## Author Contributions

TF, JS, and JW designed the study. BY, LS, CM, and JT provided information regarding the experimental design. LC, HW, and JG conducted phenotype experiments. LC, HW, and CS analyzed all the data. LC wrote the manuscript. JQ performed the RT-CPR verification. All authors read and edited the manuscript.

## Conflict of Interest Statement

The authors declare that the research was conducted in the absence of any commercial or financial relationships that could be construed as a potential conflict of interest.
